# Risk factors for heightened COVID‐19‐Related anxiety among breast cancer patients

**DOI:** 10.1002/cam4.5184

**Published:** 2022-09-04

**Authors:** Yash B. Shah, Stephanie Kjelstrom, Diana Martinez, Adam Leitenberger, Donna‐Marie Manasseh, Melissa Bollmann‐Jenkins, Ann Partridge, Virginia Kaklamani, Rowen Chlebowski, Sharon Larson, Marisa Weiss

**Affiliations:** ^1^ Sidney Kimmel Medical College Thomas Jefferson University Philadelphia Pennsylvania USA; ^2^ Breastcancer.org Ardmore Pennsylvania USA; ^3^ Main Line Health Center for Population Health Research Lankenau Institute for Medical Research Wynnewood Pennsylvania USA; ^4^ College of Population Health Thomas Jefferson University Philadelphia Pennsylvania USA; ^5^ Department of Psychiatry Columbia University Irving Medical Center New York New York USA; ^6^ Department of Surgery Maimonides Medical Center Brooklyn New York USA; ^7^ Department of Medical Oncology Dana‐Farber Cancer Institute Boston Massachusetts USA; ^8^ Division of Hematology/Oncology UT Health San Antonio San Antonio Texas USA; ^9^ The Lundquist Institute, Harbor‐UCLA Medical Center Torrance California USA; ^10^ Radiation Oncology Lankenau Medical Center Wynnewood Pennsylvania USA

**Keywords:** anxiety, breast cancer, COVID‐19, mental health, patient‐reported experiences, screening, social determinants of health

## Abstract

**Background:**

The COVID‐19 pandemic has disrupted medical care, increased isolation, and exacerbated anxiety in breast cancer patients. Since March 2020, Breastcancer.org experienced a sustained surge in requested pandemic‐related information and support. To characterize the pandemic‐related experiences of breast cancer patients, we surveyed the Breastcancer.org Community early in the COVID‐19 era.

**Methods:**

Breastcancer.org Community members were invited to complete an online questionnaire regarding their experience during the pandemic. Self‐reported data on demographics, comorbidities, care disruptions, anxiety, coping ability, telemedicine use, and satisfaction with care were collected. Results were analyzed using Stata 16.0 (Stata Corp., Inc).

**Results:**

Included were 568 current and previous breast cancer patients, primarily with U.S. residence. Overall, 43.8% reported at least one comorbidity associated with severe COVID‐19 illness and 61.9% experienced care delays. Moderate to extreme anxiety about contracting COVID‐19 was reported by 36.5%, increasing with number of comorbidities (33.0% vs. 55.4%, *p* = 0.021), current breast cancer diagnosis (30.4% vs. 42.5%, *p* = 0.011), and poorer coping ability (15.5% vs. 53.9%, *p* < 0.0001). Moderate to extreme anxiety about cancer care disruptions was reported by 29.1%, increasing with current breast cancer diagnosis (19.1% vs. 38.9%, *p* < 0.0001), actual delayed care (18.9% vs. 35.3%, *p* < 0.0001), and poorer coping ability (13.1% vs. 57.7%, *p* < 0.0001). Most utilized telehealth and found it helpful, but also expressed increased anxiety and subjectively expressed that these were less preferable.

**Conclusion:**

Early in the COVID‐19 pandemic, anxiety was reported by a large proportion of breast cancer patients, with increased prevalence in those with risk factors. Attention to mental health is critical, as emotional distress not only harms quality of life but may also compromise outcomes.

## Graphical abstract

COVID‐related anxiety is prevalent in breast cancer patients, particularly in those with a current diagnosis, co‐morbidities, care delays and disruptions. It’s important for health care providers to measure and manage pandemic‐related anxiety to improve access to care and mental health services, and to assist in COVID risk mitigation.

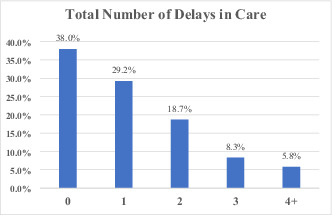

## 1 BACKGROUND

The COVID‐19 pandemic has had a profoundly negative impact on the care of breast cancer patients, whose outcomes largely depend upon early detection and timely treatment.^1^ Breast cancer patients experienced major disruptions to their care as COVID‐19 surges overwhelmed hospitals and outpatient facilities.^2^, ^3^, ^4^, ^5^ In addition, patients themselves frequently postponed or canceled scheduled visits due to fear of contracting COVID‐19 in health care settings.^1^ These compounded care delays resulted in unmanaged symptoms and pathologic upstaging,^6^ burdening patients with more intensive treatments, poorer outcomes, and increased care costs.^7^, ^8^

Conversely, a current cancer diagnosis is a significant risk factor for contracting COVID‐19 and its complications,^9^ largely due to a compromised immune system from cancer and its associated treatments.^10^, ^11^, ^12^ COVID‐19 mortality is approximately 10 times higher in cancer patients compared to non‐cancer patients.^13^, ^14^, ^15^ Moreover, recent articles have demonstrated the distinct COVID‐19‐related morbidity and mortality in cancer patients, along with the commonality of atypical symptoms, differing humoral responses to vaccination, and the suboptimal outcomes of these patients.^16^, ^17^, ^18^, ^19^, ^20^, ^21^, ^22^ Mortality risk is further compounded in cancer patients with advanced age and comorbidities including cardiovascular disease, chronic kidney disease, liver or lung disease, dementia and neurological conditions, diabetes, HIV infection, and obesity.^23^, ^24^ Although one meta‐analysis by Giannakoulis and colleagues (2020) found a minimal increase in COVID‐19 mortality for elderly cancer patients, the authors clarified that a cancer diagnosis was unlikely to significantly increase already‐high baseline risk due to advanced age and preexisting comorbidities.^24^ Importantly, these risks can significantly increase anxiety and harm mental health in patients.

Early studies showed that disruptions to care caused by the COVID‐19 pandemic increased patient anxiety and fear of cancer recurrence.^5^ Notably, a large body of literature has shown that those suffering from pandemic‐related psychological distress tend to exhibit elevated levels of post‐traumatic stress, general stress, anxiety, health anxiety, and suicidality.^25^, ^26^, ^27^ More specifically to the recent COVID‐19 pandemic, there is a syndromic disorder, COVID‐19 anxiety syndrome, associated with anxious behaviors related to the current pandemic which can be measured using the COVID‐19 anxiety syndrome scale.^28^ Social isolation, fear of infection, economic insecurity, restrictions on support persons, and other pandemic‐related challenges to daily life exacerbated the stress, trauma, and depression associated with a cancer diagnosis.^13^, ^29^, ^30^ These findings are concerning, given that a recent pre‐pandemic meta‐analysis of breast cancer patients showed that anxiety and depression serve as predictors of poorer prognosis. The authors demonstrated that these mental health symptoms were associated with increased recurrence and mortality.^31^ Thus, given the increased prevalence of depression and anxiety during the pandemic,^32^ the potential impact on cancer patients is of significant concern.

At Breastcancer.org, a leading education and support platform, widespread indicators of emotional distress among breast cancer patients and survivors were observed via online discussion boards and feedback forms during the pandemic. Support communities such as Breastcancer.org can facilitate cancer patient wellness. Thus, in order to inform patient‐targeted communications surrounding mental health and anxiety, Breastcancer.org conducted an online global survey of website visitors and online community members following the pandemic's onset in the U.S. The purpose of this study was to (1) assess the self‐reported prevalence of anxiety about contracting COVID‐19 and anxiety about care disruptions and (2) discuss the need for mental health evaluation and provide practicing oncologists with the tools to recognize these issues, ensuring their patients can be quickly referred to mental health care. Given the impact of mental health on cancer survival and the multitude of treatments available, it is crucial to recognize these symptoms, even as the pandemic cautiously eases and cancer care returns to pre‐COVID‐19 levels.

## 2 METHODS

Breastcancer.org is a nonprofit organization that provides medical expertise and a peer‐support community to a global audience of over 20 million unique users while advancing shared patient and doctor decision‐making and producing patient‐centered research. From April 27 to June 1, 2020, Breastcancer.org conducted a retrospective cross‐sectional survey where Community Members were invited to participate in an anonymous, self‐reported online questionnaire within the discussion board section of the website (Survey Monkey, Momentive, Inc.) regarding the pandemic's impact upon their care and well‐being. The survey was developed by Breastcancer.org prior to the release and validation of the COVID Anxiety Syndrome Scale (see survey tool in Appendix S2). Because this was a patient‐reported prevalence of anxiety survey study, we did not assess the syndrome specifically. This study was deemed IRB‐exempt by the institutional review committee as this project does not involve “human subjects research” as defined by Department of Health and Human Services Protection of Human Subjects regulations (45 CFR 46). Informed consent was obtained prior to survey participation. The survey and data analysis were conducted in accordance with recommendations from the Strengthening and Reporting of Observational Studies in Epidemiology (STROBE) initiative. All data were patient‐reported and duplicate data or incomplete surveys were excluded.

Patient‐reported unidentifiable data were collected on the following: demographics (including sex, age, residence, and tumor characteristics), comorbidities associated with risk of severe illness or complications from COVID‐19 infection (including lung, heart, liver or kidney disease, asthma, diabetes, obesity, and other chronic health conditions),^5^, ^9^, ^33^ provider‐initiated care delays; self‐initiated care delays, anxiety about contracting COVID‐19, anxiety about breast cancer care being disrupted by the pandemic, coping mechanisms, use of telemedicine, and satisfaction with health care services. Outcomes for anxiety about COVID‐19 infection and breast cancer care disruptions were combined into three levels: not anxious, slightly/somewhat anxious, and moderately/extremely anxious. Total comorbidities were reported as zero, at least 1, or two or more. There were few participants aged 25–34, so age categories 25–34 and 35–44 were collapsed. Similarly, coping mechanism responses were collapsed into two categories for regression analyses.

Data verification with electronic medical records was not performed. Statistical analysis was utilized to evaluate patient‐specific factors modulating anxiety levels. Data analysis was completed using Stata 16.0 (Stata Corp., Inc). Aggregate results were tabulated as frequencies and compared using chi‐square test for independence. Multivariable ordinal logistic regression was utilized to calculate associations with anxiety about contracting COVID‐19 and treatment delays. Adjusted odds ratios and 95% confidence intervals were reported. A *p*‐value of 0.05 was initially considered significant, and after Benjamini‐Hochberg correction, significance was reported accordingly. A subset of this analysis was previously presented at the 2021 ASCO Annual Meeting.

## 3 RESULTS

In total, 568 current and previous breast cancer patients from 24 countries and 43 U.S. states completed the survey (Table 1). Respondents were mostly women (99.5%) aged 55 years or older (66%) residing in the US (83.4%; Table 1), of whom 284 patients (50%) reported completing breast cancer treatment, while the remaining 284 (50%) were in active treatment or had not yet started treatment for a current diagnosis. Among all respondents, 249 (43.8%) reported having one of these comorbidities: asthma (14.3%), other lung diseases (3.9%), heart disease (6.9%), kidney disease (2.8%), liver disease (1.2%), obesity (17.1%); diabetes (7.0%); and hypertension (7.4%). Of this group, 175 (30.8%) reported one of these, 58 (10.2%) had two, 11 (1.9%) had three, and 5 (0.9%) had four of these diagnoses in addition to breast cancer.

**TABLE 1:** Demographics of survey participants

	*N* = 568
Age n (%)	
25–34	7 (1.3%)
35–44	51 (9.0%)
45–54	135 (23.9%)
55–64	184 (32.5%)
65+	189 (33.4%)
Sex n (%)	
Female	564 (99.5%)
Male	3 (0.5%)
Breast Cancer Diagnosis n (%)	
Past	284 (50%)
Current	284 (50%)
Comorbidities n (%)	
*Yes Only*	
Lung disease	22 (3.9%)
Asthma	81 (14.3%)
Heart condition	39 (6.9%)
Obesity	97 (17.1%)
Diabetes	40 (7.0%)
Kidney disease	16 (2.8%)
Liver disease	7 (1.2%)
Hypertension	42 (7.4%)
Total Comorbidities n (%)	
0	319 (56.2%)
at least 1	175 (30.8%)
at least 2	58 (10.2%)
at least 3	11 (1.9%)
4 or more	5 (0.9%)
Top 5 Countries n (%)	
United States	472 (83.4%)
United Kingdom	33 (5.8%)
Canada	21 (3.7%)
Australia	11 (1.9%)
India	5 (0.9%)
Top 5States n (%)	
California	57 (12.1%)
New York	46 (9.8%)
Pennsylvania	40 (8.5%)
Florida	33 (7.0%)
Texas	26 (5.5%)

### 3.1 Anxiety about contracting COVID‐19

Overall, 298 (57.2%) of participants reported being slightly/somewhat anxious and 206 (36.5%) were moderately/extremely anxious about contracting COVID‐19 (Table 2). Anxiety about COVID‐19 infection was more common among those with a current vs. past breast cancer diagnosis (120 [42.5%] vs. 86 [30.4%], *p* = 0.011), and those with two or more vs. no comorbidities (41 [55.4%] vs. 105 [33%], *p* = 0.002). Anxiety about contracting COVID‐19 also increased with age (*p* = 0.04): among 35‐44‐year‐olds, 13 (26.0%) reported moderate/extreme anxiety, while 85 (45.2%) respondents aged 65 or older reported the same level of distress. However, after multiple testing correction, the relationship between anxiety and age was no longer significant.

**TABLE 2:** Factors influencing anxiety about contracting COVID‐19

	Anxiety level			*p*‐value
	No anxiety	Slightly/somewhat	Moderately/extremely	
	*N* = 61 (10.8%)	*N* = 298 (52.7%)	*N* = 206 (36.5%)	
Breast cancer				**0.011** *
Past	33 (11.6)	164 (58.0)	86 (30.4)	
Current	28 (10.0)	134 (47.5)	120 (42.5)	
Comorbidities				**0.002** *
0	43 (13.5)	170 (53.5)	105 (33.0)	
1	14 (8.1)	99 (57.2)	60 (34.7)	
2+	4 (5.4%)	29 (39.2%)	41 (55.4%)	
Age				**0.04**
25–34	0 (0)	4 (57.1)	3 (42.9)	
35–44	6 (12.0)	31 (62.0)	13 (26.0)	
45–54	19 (14.1)	74 (54.8)	42 (31.1)	
55–64	25 (13.7)	96 (52.5)	62 (33.9)	
65+	11 (5.9)	92 (48.9)	85 (45.2)	
Level of coping				**<0.0001** *
I am coping very well	27 (32.1%)	44 (52.4%)	13 (15.5%)	
I am coping well	17 (8.6%)	106 (53.5%)	75 (37.9%)	
I am coping fairly well	15 (5.9%)	136 (53.8%)	102 (40.3%)	
I'm not coping well at all	2 (7.7%)	10 (38.5%)	14 (53.9%)	

Bold indicates *p* < 0.05.

* Significant after Benjamini‐Hochberg correction.

There were significantly higher odds of anxiety about contracting COVID‐19 for participants with current cancer diagnoses (aOR 1.6 [95% CI 1.2–2.3] *p* < 0.0001), with two or more comorbidities (aOR 2.2 [95% CI 1.3–3.6], *p* = 0.004), aged 65 years or older (aOR 2.1 [1.1–3.7], *p* = 0.018), and coping fairly or not well at all (aOR 1.8 [1.3–2.4], *p* = 0.001) compared to those with past diagnoses, with no other comorbidities, aged 25–44 years old, and who were coping very well or well (Table 3).

**TABLE 3:** Ordinal mulitvariable logistic regression for associations with anxiety about contracting COVID‐19

	aOR (95% CI)	*p*
Breast cancer		
Past	Ref	
Current	1.6 (1.2, 2.3)	**0.003** *
Comorbidities		
0	Ref	
1	1.1 (0.8, 1.6)	0.561
2+	2.2 (1.3, 3.6)	**0.004** *
Age		
25–44	Ref	
45–54	1.1 (0.6, 2.0)	0.73
55–64	1.1 (0.7, 2.0)	0.712
65+	2.1 (1.1, 3.7)	**0.018** *
Level of coping		
Very Well/Well	Ref	
Fairly Well/Not at All	1.8 (1.3, 2.4)	**0.001** *

Bold indicates *p* < 0.05.

* Significant after Benjamini‐Hochberg correction.

### 3.2 Delays in cancer care and associated anxiety

**FIGURE 1:**
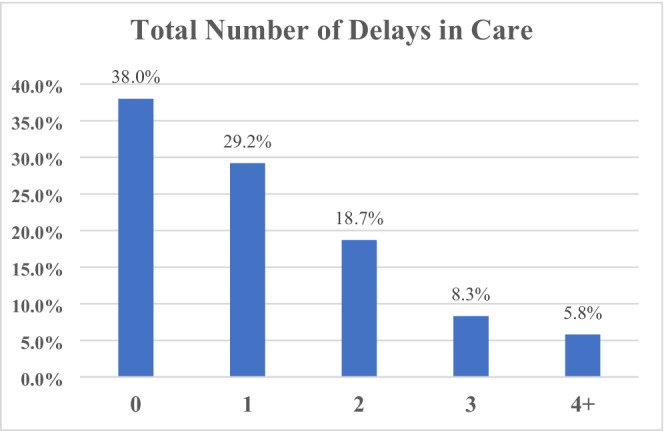
Patient‐Reported Breast Cancer Care Delays. Care delays were highly prevalent early in the pandemic and continue to persist. Among study participants, 352 (62.0%) experienced delays in oncology care that were either initiated by the patient themselves or their provider. Of those, 166 (47.1%) experienced at least one delay, 106 (30.1%) two, 47 (13.4%) three, and 33 (9.4%) four.

**FIGURE 2:**
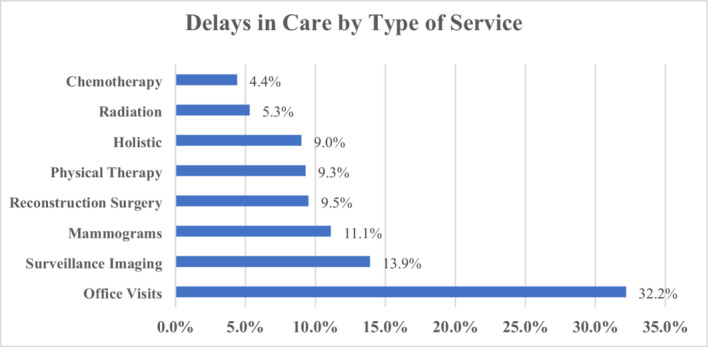
Delays in Breast Cancer Care by Type of Service. Care delays were seen in a wide variety of breast cancer care services. Delays affected office visits (32.2%), surveillance imaging (13.9%), mammograms (11.1%), reconstruction surgery (9.5%), physical therapy (9.3%), holistic services (9.0%), radiotherapy (5.3%), and chemotherapy (4.4%).

Among participants, 352 (62.0%) experienced delays in oncology care that were directly attributable to the pandemic, either initiated by the patient themselves or their provider (Figure 1). Of those, 166 (47.1%) experienced at least one delay, 106 (30.1%) two, 47 (13.4%) three, and 33 (9.4%) four. Delays affected office visits (32.2%), surveillance imaging (13.9%), mammograms (11.1%), reconstruction surgery (9.5%), physical therapy (9.3%), holistic services (9.0%), radiotherapy (5.3%), and chemotherapy (4.4%) (Figure 2).

Regarding anxiety surrounding breast cancer care disruptions, 285 (50.9%) reported slight/somewhat anxiety and 163 (29.1%) expressed moderate/extreme anxiety (Table 4). Moderate/extreme anxiety levels were significantly more common with current breast cancer diagnoses compared to past diagnoses (110 [38.9%] vs 53 [19.1%], *p* < 0.0001). Participants experiencing care delays were significantly more likely to report moderate/extreme anxiety (123 [35.3%] vs 40 [18.9%], *p* < 0.0001).

**TABLE 4:** Ordinal multivariable logistic regression for associations of anxiety about breast cancer care being affected by COVID‐19

	aOR (95% CI)	*p*
Breast cancer		
Past	Ref	
Current	**2.7 (1.9, 3.9)**	**<0.0001** *
Comorbidities		
0	Ref	
1	0.7 (0.5, 1.0)	0.055
2+	0.8 (0.5, 1.4)	0.512
Age		
25–44	Ref	
45–54	0.8 (0.5, 1.6)	0.593
55–64	0.8 (0.4, 1.5)	0.551
65+	0.6 (0.3, 1.1)	0.093
Experienced a delay in care		
Yes	**2.1 (1.4, 3.0)**	**<0.0001** *
No	Ref	
Used telemedicine		
Yes	1.3 (0.9, 1.9)	0.115
No	Ref	
Level of coping		
Well/Very Well	Ref	
Fairly Well/Not Well at All	**2.2 (1.5, 3.1)**	**<0.0001** *

Bold indicates *p* < 0.05.

* Significant after Benjamini‐Hochberg correction.

There were significantly higher odds of anxiety about care disruptions for participants with a current cancer diagnosis (aOR 2.7 (95% CI 1.9–3.9)), those who experienced a care delay (aOR 2.1 (95% CI 1.4–3.0)), and those who said they were coping fairly well or not at all with pandemic challenges (aOR 2.2 (95% CI 1.5–3.1)). Comorbidities and age group were not associated with anxiety about breast cancer care being affected by the pandemic.

### 3.3 Coping with the challenges of COVID‐19

Of the 564 participants who reported on their ability to cope with the challenges of COVID‐19, 87 (15.4%) stated they were coping very well, 198 (35.1%) coping well, 253 (44.9%) coping fairly well, and 26 (4.6%) not coping well at all. Participants with moderate/extreme anxiety about contracting COVID‐19 reported they were not coping at all or fairly well at significantly higher levels than those coping well or very well (116 [56.9%] vs 88 [43.1%], *p* < 0.0001). By contrast, those with no anxiety about contracting COVID‐19 were more likely to select coping well or very well (44 [72.1%] vs 17[27.9%]) (Table S1). In sum, poorer coping ability was associated with higher anxiety levels. Moderate/ extreme anxiety about cancer care being affected by COVID‐19 reported by 11 (13.1%) of participants who said they were coping very well, 48 (24.6%) of those coping well, 88 (35.1%) of those coping fairly well, and 15 (57.7%) of those not coping well (*p* < 0.0001).

Respondents also provided open‐ended comments about coping. Those coping better shared themes of gratitude, faith, enjoying the outdoors, and trusting safety protocols including masking and social distancing. Those with poorer coping experiences expressed themes of sadness, isolation from family, stress, anxiety about viral contraction, and the difficulties of having cancer while enduring a pandemic (Table S2).

### 3.4 Use of telemedicine during COVID‐19

331 (58.6%) had at least one virtual appointment via telephone or video, and 253 (75.3%) of these found such appointments helpful (Table S3). However, those using telemedicine reported moderate/extreme anxiety about care disruptions more frequently than those not using telemedicine (111 [34.1%] vs. 45 [25.0%], *p* = 0.010).

Similarly, in their subjective comments, many respondents found virtual appointments less preferable than in‐person appointments. Those who did not find virtual appointments helpful found their care subpar, and one participant expressed that their disease progression would have been caught earlier if not for a virtual appointment (Table S4). Nonetheless, some preferred virtual visits due to time saved.

## 4 DISCUSSION

Our study confirms previously observed disruptions in breast cancer care following the onset of the COVID‐19 pandemic and further reveals significant anxiety levels among both current and previous breast cancer patients.^30^, ^34^, ^35^ Most notably, our study demonstrates that increased anxiety levels were correlated to breast cancer patients' individual COVID‐19 risk factors. Another study found that, of all types of cancer, breast cancer patients had the highest level of COVID‐19‐related anxiety.^13^ Taken together, these findings show the relevance of mental health issues in this cohort.

### 4.1 COVID‐19 risk linked with anxiety levels in breast cancer patients

Participants currently under treatment for breast cancer or having at least 3 other comorbidities reported significantly higher COVID‐19‐related anxiety compared to those post‐treatment or without comorbidities (Table 3). These findings indicate that anxiety is proportional to the risks of complications, hospitalization, and mortality. Higher anxiety was also associated with care delays and poorer coping. This correlation between anxiety and underlying risk follows the cumulative burden and allostatic load models, where all stressors are summated to estimate the burden of anxiety. If dose–response theory is applied, the perceived level of anxiety for a patient may serve as a valuable predictor for clinicians to estimate patients' risk of COVID‐19 infection, likelihood of complications, and ultimate outcomes.^36^

While often overlooked, anxiety is an important indicator of somatic disease risk, and may play a role in patient outcomes.^37^, ^38^, ^39^, ^40^ A recent meta‐analysis that included more than 280,000 breast cancer participants reported that higher anxiety is associated with breast cancer recurrence (17% increase) and all‐cause mortality (13% increase).^31^ Although the causative link between anxiety and worse outcomes is currently unknown, assessing patient‐reported anxiety would help clinicians improve their patient's well‐being. Undoubtedly, breast cancer patients require emotional support, reliable access to providers, and increased attention to mental health.

### 4.2 Anxiety associated with cancer care disruptions

Study respondents reported limited access to oncologists and other providers, with over 60% experiencing care delays. Most respondents experienced at least one disruption, which led to significant reported anxiety about additional disruptions, which have persisted throughout the ongoing pandemic (Table S5). These findings are unsurprising given that institutions have urgently shifted resources towards the intensive care of COVID‐19 patients while postponing non‐urgent or elective appointments and surgeries.^2^, ^3^ Multiple studies reported that half of all cancer patients, and breast cancer patients in particular, experienced care disruption.^34^, ^35^ One study similarly showed about half of breast cancer patients experienced a disruption to their radiotherapy regimen.^5^

Furthermore, delays in surveillance imaging can produce significant anxiety due to the fear of worse outcomes. While the impact of delayed imaging is poorly understood, studies project an excess of cancer diagnoses and deaths over the next decade^41^; screening is estimated to reduce breast cancer mortality by 20%,^42^ but screening was paused in response to the American Cancer Society and other consortia recommendations.^43^ In fact, a 51.8%–94% decline in new diagnoses has been reported, likely affecting women with and without a history of breast cancer.^6^, ^41^, ^42^, ^44^, ^45^ An Italian study reported a rise in node‐positive and stage III breast cancer diagnoses, with fewer in situ diagnoses following a two‐month halt in mammograms.^46^ Notably, although scheduling has slowly returned to normal, this was far slower for people of color or others facing severe health disparities.^47^

In addition, many of our study respondents had disruption in their care for other comorbidities, compounding the negative impact of the pandemic on their overall care. Health care access was further limited by mounting unemployment and subsequent loss of employer‐based insurance.^42^, ^48^

### 4.3 Telemedicine alternatives to in‐person meetings

Telemedicine appointments by phone or video were offered to most study respondents as an alternative to postponed in‐person appointments, as has been widely reported during the pandemic.^5^, ^49^ The majority of survey respondents found these encounters helpful. Although telemedicine eased some care disruptions, limitations were identified. Study participants expressed significant concerns with virtual visits and provided feedback containing themes of isolation. It was reported that telemedicine was impersonal, lacking in quality, and inaccessible when lacking reliable internet or technological skill. However, this participant feedback was collected at the start of the pandemic when access to and functionality of telemedical care was relatively limited. A 2020 study indicated signs of progress, with greater provider training and experience,^50^ improved institutional participation^51^, ^52^ and new infrastructure to increase patient accessibility.^49^, ^53^ It is possible that with improvements, telemedicial care can offer great benefit to patients after the pandemic. As COVID‐19 was not the first global pandemic and will likely not be the last, better preparation of telemedicine services is an area of opportunity for future mental health care.

The value of in‐person visits with doctors was also hampered by the pandemic, as many patients received care alone. Family and friends were often barred from attending medical appointments to reduce transmission risk, missing the opportunity to visit inpatients or provide support during consultation, surgery, chemotherapy, or radiotherapy.^29^, ^54^ Moreover, pandemic constraints made it difficult for patients' immediate support networks to assist with everyday demands including childcare or food. The ensuing feelings of abandonment^30^, ^43^, ^55^ caused additional distress. Prolonged loneliness has been shown to increase depression and anxiety while worsening quality of life for breast cancer patients,^29^, ^30^, ^54^ increasing the negative impact of cancer and the pandemic on mental health.

### 4.4 Next steps

For the clinician, our study findings, combined with previous literature, highlight the utility of mental health evaluation in informing cancer prognosis and assisting with treatment of anxiety and poor coping skills. The National Cancer Institute provides updates and guidelines that can be used to assess cancer patients.^56^ This website recommends using the National Comprehensive Cancer Network Distress Thermometer to screen for patients needing mental health services.^57^ This one‐page questionnaire is filled out by the patient and uses a simple scale of 1–10 assess distress. Patients scoring ≥7 should undergo further thorough psychosocial evaluation.^58^ Additional tools that can be used to assess symptoms include the General Health Questionnaire (GHQ‐12), the Beck Depression Inventory, and the Beck Anxiety Inventory, all of which are readily available online.^59^, ^60^, ^61^ Self‐assessment tools are also featured on Breastcancer.org

Remedies for anxiety and depression include a wide range of options. Some cancer patients will improve with psychoeducational support groups,^62^ online communities and digital tools,^63^, ^64^, ^65^ yoga,^66^ or mindfulness‐based interventions,^67^ including internet‐delivered mindfulness‐based cognitive therapy.^68^ Other patients may require more intensive psychotherapy or pharmacotherapy, including benzodiazepines and anti‐depressants. Guidelines on the treatment of anxiety and depression of adults with cancer have been previously published.^69^ Clinicians may even target anxiety by reducing treatment interruptions and improving the patient experience through a variety of changes, such as easier scheduling, less waiting times, increased employment of oral chemotherapies, and utilization of shorter breast radiotherapy hypofractionation schedules.^70^, ^71^ Certainly, clinical applications depend on the uniqueness of each community, ethno‐racial group, culture, and geographic region.

Although COVID‐19 was not the first global pandemic, the recent growth in social media and telemedicine offered great opportunity for improved patient services for future crises. While mental health services were not well‐developed in advance of this pandemic, the lessons learned from this experience may inform better care for future patient needs.

In summary, the evaluation of anxiety, depression, and other symptoms of mental illness may alert clinicians to key patient‐specific risk factors which may affect their cancer prognosis and treatment planning. Taking these steps will confer superior management of anxiety and mental distress while improving utilization of breast cancer screening, therapy, and follow‐up care, with or without a concurrent public health crisis.

### 4.5 Limitations

Our study primarily captured a U.S. nationally representative sample of breast cancer patients to provide real‐time insights into their care experiences and assess their needs and vulnerabilities. The survey completion rate was 99.8%. While online surveys carry inherent limitations including risks of recall and selection biases, online convenience sampling has become a critically important research tool to obtain a rapid snapshot of a rapidly changing situation, from a geographically diverse population, while conventional study methods remain constrained by the pandemic.^30^, ^72^ Importantly, given this format, clinical mental health diagnoses could not be made, and all data were patient‐reported, which provides some benefits in everyday use but may limit accuracy.

The lack of verification of patient‐reported data is a limitation. For example, in our study, patient‐reported frequency of obesity of only 17.1% is likely inaccurate considering the substantially higher U.S. prevalence of obesity of 42.4%.^73^ Self‐reported obesity may be inaccurate for various reasons including respondents' inability to accurately self‐classify utilizing established BMI thresholds.^74^ Obesity underreporting may weaken the link between anxiety levels and comorbidities, given its prevalence in the U.S. and associated high risk of severe illness and complications from COVID‐19 infection.^23^

Another important limitation is a lack of demographic data capturing respondents' race, ethnicity, socioeconomic status, social support, or education level, given the impact of these factors on anxiety and COVID‐19 outcomes.^75^ For instance, Black cancer patients have a higher risk of poorer COVID‐19 outcomes among those recently diagnosed.^9^ An additional limitation of our study is the absence of patients' COVID‐19 infection diagnosis status, which is associated with greater severity in patients with current cancer diagnosis and can complicate cancer treatment side effects and worsens social isolation.

While respondents' reported anxiety levels were captured, diagnosed anxiety and fear‐related disorders were not included as comorbidities of interest, as they were not established COVID‐19 risk factors when this survey was conducted. However, subsequent research has since established that diagnoses of anxiety and fear‐related disorders are principal risk factors for severe COVID‐19 illness,^33^ reinforcing the urgency of cancer patient mental health screening and referral to counseling, as well as full risk assessment and reduction. The absence of a general population control group limits the study's ability to determine if the signals of increased anxiety in breast cancer patients with comorbidities might also be observed in other populations. A lack of control alongside varying survey response rates may imply sampling bias, although previous patient‐reported mental health and breast cancer studies, along with STROBE recommendations, have clarified this issue.^76^

Finally, this study was conducted at the onset of the pandemic and its findings may not reflect the patient experience at later times of the pandemic. Nonetheless, this study offered an early insight into the pandemic and directly captured patient feedback, offering a unique and timely look at ongoing challenges in oncology.

## 5 CONCLUSIONS

This Breastcancer.org Community survey provides key insights into the widespread distress experienced by breast cancer patients worldwide during the onset of the pandemic. Anxiety, an indicator of emotional distress, was positively associated with risk factors for serious COVID‐19 infection. Major disruptions of care and social isolation, including reduced access to providers, appointment delays and loss of social support systems were all associated with increasing levels of anxiety. Although often overlooked, anxiety can have a profound negative effect on quality of life and worse outcomes following both COVID‐19 and cancer diagnoses.

This study demonstrates that breast cancer patients are a vulnerable population that can benefit from improved mental health services, tailored COVID‐19 risk assessment, and implementation of systematic care improvements and precautions. Healthcare providers should encourage their patients to report the quality of their cancer care experience, including their emotional and physical signs and symptoms and any barriers to accessing their providers, as well as their COVID‐19 risks, safety precautions, and vaccination status.

## AUTHOR CONTRIBUTIONS

**Yash B. Shah:** Conceptualization (equal); formal analysis (equal); investigation (equal); writing – original draft (equal); writing – review and editing (equal). **Stephanie Kjelstrom:** Formal analysis (equal); methodology (equal); visualization (equal). **Diana M. Martinez:** Project administration (equal); writing – original draft (equal); writing – review and editing (equal). **Adam Leitenberger:** Methodology (equal); project administration (equal). **Donna Marie‐Manasseh:** Writing – review and editing (equal). **Melissa Bollmann‐Jenkins:** Writing – review and editing (equal). **Ann Partridge:** Resources (equal); writing – review and editing (equal). **Virginia G. Kaklamani:** Validation (equal); writing – review and editing (equal). **Rowan T Chlebowski:** Validation (equal); writing – review and editing (equal). **Sharon L. Larson:** Formal analysis (equal); investigation (equal); supervision (equal); visualization (equal); writing – review and editing (equal). **Marisa Carey Weiss:** Conceptualization (equal); investigation (equal); methodology (equal); project administration (equal); writing – review and editing (equal).

## FUNDING INFORMATION

The effort of DM is supported by federal grant K24 DA‐20620‐05. All other authors report no funding sources.

## CONFLICT OF INTEREST

The authors have no conflicts of interest to declare. The authors were entirely responsible for the design and conduct of the study; the analysis and interpretation of the data; and preparation, review, approval, and submission of the manuscript for publication.

## ETHICS AND INSTITUTIONAL REVIEW BOARD APPROVAL

This study was deemed IRB‐exempt as this project does not involve “human subjects research” as defined by the Department of Health and Human Services Protection of Human Subjects regulations (45 CFR 46). No identifiable information was collected from respondents. Informed consent was obtained.

## Supporting information


Appendix S1

Appendix S2

## Data Availability

The data that support the findings of this study are available from the corresponding author upon reasonable request.
