# Supplementary material for: Risk factors for heightened COVID‐19‐Related anxiety among breast cancer patients
Source: Cancer Med. 2022 Sep 4;12(3):3577–88. doi: 10.1002/cam4.5184 (PMC9538212; doi:10.1002/cam4.5184)
Supplement: Supplementary file 1 — Appendix S1 [file CAM4-12-3577-s001.pdf]

Has COVID-19 Affected Your Breast Cancer Care?

COVID-19 has changed virtually every aspect of our lives, especially our healthcare. With certain cancer treatments being delayed or changed, this can be a particularly distressing time for people who have been diagnosed with cancer.

To learn more about how COVID-19 is impacting breast cancer care, we need to hear from you. Please complete the survey below to help us understand how COVID-19 has affected your breast cancer treatment, screening/surveillance, or survivorship.

Thank you in advance for helping us to better serve the breast cancer community. The survey should take between 5-10 minutes to complete.

1. What is your age?

- ◯ 18-24
- ◯ 25-34
- ◯ 35-44
- ◯ 45-54
- ◯ 55-64
- ◯ 65+

2. What is your gender?

- ◯ Male
- ◯ Female
- ◯ Other

3. In what country do you live?

4. In what state do you reside if living in the United States?

5. What best describes your breast cancer history?

- ◯ Diagnosed and in active treatment
- ◯ Diagnosed and have not yet started treatment
- ◯ Diagnosed in the past, completed treatment, and receiving routine surveillance
- ◯ Found a lump or had suspicious/abnormal imaging but have not yet received a biopsy or biopsy results
- ◯ Never diagnosed but receiving increased screening due to family history or high risk
- ◯ Never diagnosed and average risk (receiving standard annual mammogram)
- ◯ Caregiver to someone with breast cancer (if you select this option, please answer the following questions about your loved one's treatment)
- ◯ Other (please specify)

6. Have you been diagnosed with metastatic/stage IV breast cancer?

- ◯ Yes
- ◯ No
- ◯ Other (please specify)

7. Have you developed COVID-19?

- ◯ Yes, I tested positive
- ◯ Yes, a doctor diagnosed me but I was not tested
- ◯ I developed symptoms but was not diagnosed by a doctor
- ◯ I had some symptoms but I’m not sure it was COVID-19
- ◯ I thought I had symptoms but I tested negative
- ◯ I have not experienced symptoms and I have not been tested

8. Do you have any of the following health conditions?

- [ ] Lung disease
- [ ] Asthma
- [ ] A heart condition
- [ ] Obesity
- [ ] Diabetes
- [ ] Kidney disease
- [ ] Liver disease
- [ ] HIV/AIDS
- [ ] Organ transplant

Other chronic health condition [Specify]

9. Have any aspects of your breast cancer screening/surveillance, treatment, or follow-up been **delayed or changed by your healthcare provider** because of COVID-19?

Please select as many options as necessary.

- [ ] Routine annual mammogram
- [ ] Surveillance imaging (follow-up scans if you've been previously diagnosed)
- [ ] Biopsy
- [ ] Lumpectomy
- [ ] Mastectomy
- [ ] Reconstruction
- [ ] Hormonal therapy
- [ ] Radiation therapy
- [ ] Targeted therapy
- [ ] Chemotherapy
- [ ] Immunotherapy
- [ ] Any form of palliative care
- [ ] Clinical visits (check up appointment)
- [ ] Clinical trial participation
- [ ] Mental health services
- [ ] Physical therapy
- [ ] Any complementary or holistic therapy (acupuncture, chiropractor, etc.)
- [ ] No aspects of my treatment are being delayed right now
- [ ] Not applicable
- [ ] Other (please specify)

10. Have **you chosen to delay** any aspects of your breast cancer screening/surveillance, treatment, or follow-up because of COVID-19?

Please select as many options as necessary.

- [ ] Routine annual mammogram
- [ ] Surveillance imaging (follow-up scans if you’ve been previously diagnosed)
- [ ] Biopsy
- [ ] Lumpectomy
- [ ] Mastectomy
- [ ] Reconstruction
- [ ] Surgery
- [ ] Hormonal therapy
- [ ] Radiation therapy
- [ ] Targeted therapy
- [ ] Chemotherapy
- [ ] Immunotherapy
- [ ] Any form of palliative care
- [ ] Clinical visits (check up appointment)
- [ ] Clinical trial participation
- [ ] Mental health supportive services
- [ ] Physical therapy
- [ ] Any complementary or holistic therapy (acupuncture, chiropractor, etc.)
- [ ] I have not chosen to delay any aspects of my treatment
- [ ] Not applicable
- [ ] Other (please specify)

11. If you have chosen to delay screening/surveillance, treatment, or follow-up visits, did your doctor support your decision?

- ○ Yes
- ○ No
- ○ Not applicable

12. Are you currently deciding whether to delay or cancel any upcoming medical appointments related to your breast cancer care or breast health?

- ◯ Yes
- ◯ No

13. If you have chosen to delay, or are considering changing any aspect of your care, was/is your decision-making influenced by (check all that apply):

- ☐ Concerns about contracting COVID-19
- ☐ Travel restrictions to your preferred hospital
- ☐ Policy changes at your healthcare facility
- ☐ Financial concerns
- ☐ Your doctor’s recommendation
- ☐ Your family’s/friends’ advice
- ☐ Not applicable
- ☐ Other (please specify)

14. If you are currently receiving treatment for breast cancer, has your treatment been relocated to a different healthcare facility from where you normally receive it?

- ◯ Yes
- ◯ No
- ◯ Not applicable

15. Has COVID-19 influenced your desire or ability to seek a second opinion at this time?

- ◯ Yes
- ◯ No
- ◯ Not applicable

16. How anxious are you generally feeling about your breast cancer care being affected by the pandemic at this time?

- Not at all anxious
- Slightly
- Somewhat
- Moderately
- Extremely

Feel free to explain:

17. How anxious are you about contracting COVID-19?

- Not at all anxious
- Slightly anxious
- Somewhat anxious
- Moderately anxious
- Extremely anxious

Feel free to explain:

18. How do you think you are coping with the challenges of COVID-19?

- I'm not coping well at all
- I am coping fairly well
- I am coping well
- I am coping very well

Feel free to explain:

19. Have any of your medical appointments been done remotely/virtually by telephone or video during the COVID-19 pandemic?

- Yes
- No
- Not applicable

20. Did you find remote/virtual appointments by phone or video to be helpful and effective?

- ◯ Yes
- ◯ No
- ◯ Not applicable

Feel free to explain:

21. During this time of the COVID-19 pandemic, how are you staying in touch with your support person(s)? (select all that apply)

- ☐ Telephone calls
- ☐ Video conferencing (e.g. via Skype, Zoom or FaceTime)
- ☐ Email
- ☐ Social Media (e.g. Facebook, Twitter, Instagram, Snapchat, or TikTok)
- ☐ Text messaging, WhatsApp or Telegram
- ☐ Other (please specify)

22. Has a healthcare provider talked with you about how a life-threatening COVID-19 infection would be managed if you were to become sick?

- ◯ Yes
- ◯ No

23. How satisfied are you with the care you are getting from your healthcare providers at this time?

- ◯ Very satisfied
- ◯ Satisfied
- ◯ Neither satisfied nor dissatisfied
- ◯ Dissatisfied
- ◯ Very dissatisfied

Feel free to explain:

24. Have you or any members of your immediate family lost their jobs due to the COVID-19 pandemic?

- ◯ Yes
- ◯ No

Feel free to explain:

25. Have you or any members of your immediate family had their hours reduced due to the COVID-19 pandemic?

- ◯ Yes
- ◯ No

Feel free to explain:

26. Has COVID-19 affected your medical insurance coverage for treatments or consultations?

- ◯ Yes
- ◯ No

Feel free to explain:

27. Has the financial impact of COVID-19 affected your ability to pay for care?

- ◯ Yes
- ◯ No

Feel free to explain:

28. Have you personally experienced any shortages of medication specifically for breast cancer treatment or symptom palliation (e.g. pain medications) because of COVID-19?

- ◯ Yes
- ◯ No

Feel free to explain:

29. If you are attending clinic/hospital appointments right now, have your transportation options been affected because of COVID-19?

- ◯ Yes
- ◯ No

Feel free to explain:

30. Due to COVID-19, are you having difficulty affording food and supplies in order to stay at home to avoid contracting the virus?

- ◯ Yes
- ◯ No

Feel free to explain:

[ ]

31. Where are you getting information about COVID-19 to help make your breast cancer treatment decisions (check all that apply)?

- ☐ Healthcare provider
- ☐ Breastcancer.org content and discussion boards
- ☐ Other breast cancer advocacy organizations
- ☐ Other medical websites
- ☐ Social media
- ☐ Government agency (CDC, FDA, WHO)
- ☐ TV News

Please specify organizations, websites and agencies here, if applicable:

[ ]

32. Share your email address so we can send you survey results and any relevant content.

Email Address [ ]

Thank you for taking the time to complete the survey.
Please contact us at comments@breastcancer.org with any questions or feedback.
