# Supplementary material for: Risk factors for heightened COVID‐19‐Related anxiety among breast cancer patients
Source: Cancer Med. 2022 Sep 4;12(3):3577–88. doi: 10.1002/cam4.5184 (PMC9538212; doi:10.1002/cam4.5184)
Supplement: Supplementary file 2 — Appendix S2 [file CAM4-12-3577-s002.docx]

**Supplement Section**

**Supplementary Table 1.** Level of Coping with COVID-19 Challenges

|  | Fairly Well /Not at All | Well /Very Well | P |
| --- | --- | --- | --- |
|  | N = 279 | N = 285 |  |
| **Breast Cancer n (%)** |  |  | 0.353 |
| Past | 133 (47.5%) | 147 (52.5%) |  |
| Current | 146 (51.4%) | 138 (48.6%) |  |
| **Comorbidities n (%)** |  |  | **0.05** |
| 0 | 142 (44.9%) | 174 (55.1%) |  |
| 1 | 95 (54.6%) | 79 (45.4%) |  |
| 2+ | 42 (56.8%) | 32 (43.2%) |  |
| **Age n (%)** |  |  | 0.773 |
| 25-34 | 3 (42.9%) | 4 (57.1%) |  |
| 35-44 | 29 (56.9%) | 22 (43.1%) |  |
| 45-54 | 63 (46.7%) | 72 (53.3%) |  |
| 55-64 | 88 (48.6%) | 93 (51.4%) |  |
| 65+ | 95 (50.5%) | 93 (49.5%) |  |
| **Anxious About** |  |  |  |
| **Contracting COVID-19**  **n (%)** |  |  | **<.0001** |
| Not Anxious | 17 (27.9%) | 44 (72.1%) |  |
| Slightly/Somewhat | 146 (49.3%) | 150 (50.7%) |  |
| Moderate/Extremely | 116 (56.9%) | 88 (43.1%) |  |
| **Anxious about breast cancer** |  |  |  |
| **care being affected by COVID-19 n (%)** |  |  | **<.0001** |
| No Anxiety | 38 (33.9%) | 74 (66.1%) |  |
| Slightly/Somewhat | 136 (48.2%) | 146 (51.8%) |  |
| Moderate/Extremely | 103 (63.6%) | 59 (36.4%) |  |
| **Experienced a Delay in Care n (%)** |  |  | 0.172 |
| Yes | 181 (51.7%) | 169 (48.3%) |  |
| No | 98 (45.8%) | 116 (54.2%) |  |
| **Used Telemedicine n (%)** |  |  | 0.134 |
| Yes | 170 (52.0%) | 157 (48.0%) |  |
| No | 82 (45.1%) | 100 (55.0%) |  |

**Supplementary Table 2.** Qualitative Analysis from Comments on Coping with the Challenges of COVID-19

| **Comments on Coping with the Challenges of COVID-19** |
| --- |
| "Have God, my family, and my health" |
| "I have a garden and the weather has been fine. I like reading. I take a good walk daily with my dog." |
| "When I do have to go out I wear a mask...But I haven't had physical contact with any family members for months, and that is the tough part." |
| "It is torture to be diagnosed with breast cancer during the pandemic. But I have found that I'm coping somewhat okay." |
| "The isolation and health concern is very stressful" |
| "It is a very scary thing...I might catch it no matter what I do to protect myself" |
| "I can't see or travel to family. Cancer is robbing me of my future and covid is robbing me of my present." |
| "Being a Cancer Patient it's difficult to stay away from adult children n Grandkids...this disease is hard n having to make oneself stay isolated is depressing." |

**Supplementary Table 3.** Factors Influencing Anxiety about Breast Cancer Care Disruptions due to COVID-19

|  | Anxiety Level | | |  |
| --- | --- | --- | --- | --- |
|  | No Anxiety | Slightly/Somewhat | Moderately/Extremely | P-value |
|  | N=112 (20%) | N=285 (50.9%) | N=163 (29.1%) |  |
| **Breast Cancer** |  |  |  |  |
| Past | 81 (29.2%) | 143 (51.6%) | 53 (19.1%) | **<.0001** |
| Current | 31 (11.0%) | 142 (50.2%) | 110 (38.9%) |  |
|  |  |  |  |  |
| **Comorbidities** |  |  |  |  |
| 0 | 58 (18.4%) | 159 (50.3%) | 99 (31.3%) | 0.101 |
| 1 | 39 (22.5%) | 96 (55.5%) | 38 (22.0%) |  |
| 2+ | 15 (21.1%) | 30 (42.3%) | 26 (36.6%) |  |
|  |  |  |  |  |
| **Age** |  |  |  |  |
| 25-34 | 0(0) | 4(57.1) | 3(42.9) | 0.097 |
| 35-44 | 4 (7.8%) | 30 (58.8%) | 17 (33.3%) |  |
| 45-54 | 22 (16.3%) | 69 (51.1%) | 44 (32.6%) |  |
| 55-64 | 40 (22.0%) | 86 (47.3%) | 56 (31.0%) |  |
| 65+ | 46 (25.1%) | 94 (51.4%) | 43 (23.5%) |  |
|  |  |  |  |  |
| **Experienced a Delay in Care** |  |  |  | **<.0001** |
| Yes | 46 (13.2%) | 179 (51.4%) | 123 (35.3%) |  |
| No | 66 (31.1%) | 106 (50.0%) | 40 (18.9%) |  |
|  |  |  |  |  |
| **Used Telemedicine** |  |  |  | **0.010** |
| Yes | 48 (14.7%) | 167 (51.2%) | 111 (34.1%) |  |
| No | 44 (24.4%) | 91 (50.6%) | 45 (25.0%) |  |
|  |  |  |  |  |
| **Level of Coping** |  |  |  | **<.0001** |
| I am coping very well | 34 (40.5%) | 39 (46.4%) | 11 (13.1%) |  |
| I am coping well | 40 (20.5%) | 107 (54.9%) | 48 (24.6%) |  |
| I am coping fairly well | 36 (14.3%) | 127 (50.6%) | 88 (35.1%) |  |
| I am not coping well at all | 2 (7.7%) | 9 (34.6%) | 15 (57.7%) |  |

**Supplementary Table 4.** Comments on Using Telemedicine During COVID-19

| **Examples of Comments about Virtual Appointments** | |  |  |
| --- | --- | --- | --- |
| 1. Those who answered "yes" virtual appointments were helpful | | | |
| "Getting the cancer diagnosis by telephone was surprising, but better than being delayed." | | | |
| "I didn't get the hands-on exam I usually get." | | | |
| "They are okay, but I really miss the ‘human touch’ " | | | |
| "It's the ONLY time I didn't have to wait 2 hours for my (oncologist)!" | | | |
|  |  |  |  |
| 2. Those who said "no" virtual appointments were not helpful | | | |
| "I don't feel like I connected with the doctor and I don't feel like he gave me the attention I would get in person." | | | |
| "I might as well treat myself!!" | | |  |
| "I have progression of my primary tumor that was missed (because) my April appt was virtual." | | | |
| "Televisit with medical provider didn’t give me the same level of care that I usually feel I get face-to-face." | | | |

|  | | | | |
| --- | --- | --- | --- | --- |
| **Supplementary Table 5.** Comparison of Two Anxiety Questions | | | | |
| Anxiety about |  |  |  |  |
| Breast Cancer | Anxiety about Contracting COVID-19 | | |  |
| Care Affected | No Anxiety | Slightly/Somewhat | Moderately/Extremely |  |
| No Anxiety | 31 (27.7%) | 56 (50.0%) | 25 (22.3%) |  |
| Slightly/Somewhat | 17 (6.0%) | 189 (66.8%) | 77 (27.2%) |  |
| Moderate/Extremely | 13 (8.0%) | 49 (30.1%) | 101 (61.9%) |  |

**Supplemental Figure 1.** Full survey tool appended.
